# Cincinnati Medical Society

**Published:** 1879-06

**Authors:** 


### CINCINNATI MEDICAL SOCIETY.

Dr. Murphy at a regular meeting of this society exhibited two kidneys, which presented in a marked degree granular degeneration. They were very small, tough, with diminished cortical substance and contained several cysts. They were removed from the body of a patient who died in the Cincinnati Hospital. She had been more than once in the hospital, the last time suffering with a moderate amount of œdema of legs and very distressing dyspnœa; her heart was much hypertrophied, but there were no indications of valvular disease. She had passed a fair amount of urine, which contained albumen, but not in large quantity. Her symptoms had been relieved to a certain extent by hypodermic injections of pilocarpin and morphia. As the post mortem was made without consent of the friends, the heart was not examined.

The doctor referred also to some other cases of Bright's disease, which he had in his wards at the present time.

One, a case probably of amyloid degeneration of the kidney, is a woman who had had syphilis with nodes on the shins, ulna and bones of the head. She had been relieved of these by the use of iodide of potassium, and had left the house. A week ago while on the street she had a convulsion and was brought into the hospital; several convulsions followed, and she became semi-comatose, from which condition she was aroused by the use of pilocarpin and drastic purgatives. However, the conditions returned and she is now insane.

Another case, chronic tubal nephritis occurring in an opium eater. When admitted the amount of urine was four ounces in twenty-four hours. She had convulsions. Pilocarpin was administered, and the convulsion ceased. She is now partially conscious and is affected with carpo-pedal spasms. She is taking bitartrate of potassium.

Another case, acute tubal nephritis. A woman seized in the evening with rigors, followed by general dropsy. When admitted she passed five ounces of urine in the twenty-four hours and was suffering with nausea. She was treated with jaborandi, and the nausea and dropsy are disappearing and the urine increasing in quantity. The doctor remarked upon the difficulty of accounting for the dropsy in those cases of Bright's disease where the urine is abundant, as in granular and amyloid kidneys.

Dr. Mackenzie stated that the case first reported, of which the kidneys had been exhibited, had been in the hospital during his term of service. When first admitted there was but little œdema, but pronounced dyspnœa. On physical examination the heart was found enlarged and a very distinct mitral systolic murmur was heard. Although albumen was found in the urine, the dyspnœa was rather referred to the cardiac difficulty. She left the hospital, very much improved as far as her breathing was concerned, and presented herself at the Miami College Dispensary. On examination then it was found that the murmur was no longer to be heard, the hypertrophy being quite as marked as before, and the urine containing albumen. The case was interesting as showing the intimate relation existing between cardiac hypertrophy and granular kidney, in further illustration of which another case was related by Dr. Mackenzie. A woman about fifty years of age presented herself at the Dispensary about three years ago. She was then complaining of some shortness of breath on exertion, and stated that she was passing more urine than before; she also had a cough. He heart was found greatly hypertrophied, but no murmur could be detected. Her urine was pale, of low specific gravity and contained no albumen, notwithstanding which, in the absence of any indications of vulvular disease, it was regarded as probable that the case was one of chronic Bright's disease, and such proved to be the case, for subsequently, though not constantly, for some time small quantities of albumen were found in the urine. She continued to come to the Dispensary at intervals for more than two years, at the end of which time she was quite dropsical and her urine was always markedly albuminous. Last December she entered the hospital, and soon after died, and her kidneys were found upon post-mortem to be markedly granular. In this case the only symptom, when she first applied for treatment, pointing in the true direction, was the cardiac hypertrophy.

Dr. John Davis thought that great benefit may follow the administration of jaborandi in these cases by eliminating water and solid materials from the system by the skin, and that, with careful administration, prostration or other disagreeable symptoms are not likely to result. He was of the opinion, that in many of the cases of so-called hypertrophy, inflammatory disease of the heart was present, and called attention to the possibility of confusing hypertrophy with effusion, the result of pericarditis, serous inflammation being not at all uncommon in Bright's disease, and also the danger of mistaking œdema of the lung for pneumonia.

Dr. Mackenzie stated that the hypertrophy of the heart in the two cases to which he had referred, was established not only by the increase of precordial dullness, but by the greatly increased area of cardiac impulse.

Dr. Murphy remarked that dropsy was not necessarily present in all cases of Bright's disease; that it was most marked in the inflammatory form, least so and sometimes absent in the cirrhotic and amyloid. Mentioned a case in his practice in which there was uræmic coma, resulting fatally without any preceding dropsy or convulsions. There are three forms of Bright's disease. Inflammatory, including the acute and chronic, cirrhotic and amyloid. These forms may be combined, but it is uncertain whether there is any relation of cause and effect between them. Hypertrophy of the heart and albuminuric retinitis, are almost distinctive of the cirrhotic form.

Dr. John Davis did not regard hypertrophy of the heart as of such frequent occurrence in Bright's disease as pericarditis with effusion, nor did he think that an increased area of impulse sufficient to justify a diagnosis of hypertrophy. He believes that dropsy is present in some stage of all cases of the disease, and that a case should not be considered one of Bright's disease unless dropsy have formed one of the symptoms at some period of its course. He did not see any reason for limiting the number of forms of the disease to three. Rokitansky described eight varieties, and other authorities state the forms as between three and eight. Referred to the occurrence of Bright's disease consequent upon irritation of the cutaneous surface, mentioning in illustration a case which had come under his observation. A woman suffering with pain in the side, for which a blister had been applied by a physician. When he saw the case shortly afterwards, the face and hands were very much swollen and the urine albuminous. Albuminuria may also be caused by turpentine applied to the skin, and Hughes Bennett relates a case in which the application of sulphur ointment for the relief of itch was followed by Bright's disease.

Dr. Comegys said that the cardiac hypertrophy may be due to difficulty in the capillary circulation, owing to defective nutrition of the nervous system from excessive drain of albumen from the blood; or it may be referred to the muscular re-action from the presence of excessive amount of excrementitious substances in the blood. He differed from Dr. Davis in regard to the significance of increased cardiac impulse. If the pericardium be distended with fluid, the impulse would be diminished instead of increased. He regarded the rapid supervention of Bright's disease as the cause of sudden death in many acute diseases as scarlatina and diphtheria, and thought that it was not sufficiently dwelt upon as a serious complication in these diseases as well as in women in the puerperal condition; the non-elimination of excrementitious substances in these cases frequently causes collapse, and the symptoms may be occasionally relieved by flushing the kidney tubes. The fatal result in yellow fever may frequently be referred to the same cause.

The order of the evening, the discussion of the alleged antagonism of morphia and atropia, was then taken up.

Dr. Comegys stated that he regarded the antagonism of these agents as pretty well established, and not only within the narrow limits maintained by Dr. W. B. Davis, but in the most marked degree. Some cases had lately come to his knowledge supporting this view. A child, suffering with some ocular disease, had prescribed for it a collyrium of atropia, and iron to be taken internally. By mistake a teaspoonful of the atropia solution was given. Symptoms of atropia poisoning soon set in; $\frac{1}{4}$ grain of morphia was injected hypodermically, and soon after another $\frac{1}{4}$ grain. The child recovered. In another case a woman took a lethal dose of atropia; the physician injected 1 grain morphia hypodermically, and the woman's life was saved. In the third case a child took a dose of morphia and was soon narcotized; $\frac{1}{2}$ grain sulphate of atropia was injected subcutaneously, and this was followed by another injection of $\frac{1}{4}$ grain. The child recovered. The quantity of morphia in this last case was not exactly known, but the symptoms of narcosis were quite pronounced. These cases occurred in the practice of Drs. Dunlap and C. O. Wright, to whom he was indebted for the particulars.

Dr. Stanton remarked upon the comparative immunity of children to the action of atropia, and their impressibility to opium. He thought that the quantity of morphia injected in one of the cases would have been fatal if it had not been preceded by the atropia.

Dr. John Davis reported the case of a man treated by him in the Cincinnati Hospital. The patient had taken one-half ounce of tincture of opium. He was treated with seven-eighths to one-quarter grain atropia injected hypodermically every two hours; electricity was also applied. The man died, the treatment seemed to have but little influence.

Dr. W. B. Davis regarded the reports as given to-night unreliable, as not being sufficiently detailed. Too much with reference to this subject has been stated loosely; and the effects of other treatment employed simultaneously not sufficiently considered; in order to be conclusive, other modes of treatment should be dispensed with. The majority of the authorities capable of judging of this matter, believe that the weight of evidence is opposed to the antagonism; the physiological effects of the two agents would not support the views enunciated by Dr. Comegys, and it must be recollected that in children as much as gr. j of atropia has been recovered from without any treatment. In his opinion atropia should be given in morphia poisoning only for the purpose of stimulating respiration and a failing heart, and if it fails in producing these effects it is injurious as intensifying the narcotic influence of the opium.

Drs. Walker and John Davis concurred with Dr. W. B. Davis in considering the details furnished by Dr. Comegys in the relation of his cases as quite insufficient in establishing any antidotal effect between the medicines under discussion, and hoped that the doctor would report them in more detail at some future time, which Dr. Comegys promised to do.

Dr. Murphy reported the following case: About two weeks ago a man was brought to the office. Up to August 28, 1878, he had always been in the enjoyment of good health. On that day he had heen seized with a slight chill, followed by a sharp fever, frequency of respiration, cough and rusty expectoration. For this he was blistered over the upper lobe of right lung. He recovered and was able to be out, but always since then has suffered with shortness of breath on exertion. He was fairly nourished; had a good appetite; slept well; could lie on either side, but only for two hours on the left side. The physician in the country, by whom he was brought here, found on measurement the right side of chest 1½ inches larger than the left, and this was confirmed; the right side was motionless on inspiration; there was absence of fremitus; absolute flatness on percussion, and very feeble respiratory murmur on right side as high as the spine of the scapula posteriorly and the third rib anteriorly. Thirty ounces of straw-colored fluid were removed by aspirator, the needle being introduced an inch below the inferior angle of the scapula. Tonics and good nutritious food were ordered, and counter-irritation by means of tincture of iodine.

Dr. Goode asked if an ordinary trochar and canula fitted to an exhausting apparatus would not be better than the needle of an aspirator. He thought there was danger of causing pain and wounding the lung as its surface came in contact with the sharp extremity of the needle after the withdrawal of the fluid.

Dr. Murphy thought that it did not make much difference whether a trochar and canula or a needle were employed. The injury to the lung might be considered as trivial, and as likely to occur with the one as the other. Never had any bad effects except the pain and shortness of breath as the fluid is withdrawn. In his case the lung was pushed up quite out of danger of puncture.

Dr. Murphy reported a case of erysipelas in a woman 50 years of age. The case, as far as the swelling and constitutional disturbance indicated, was a mild one, and seemed to yield to treatment with tonics and stimulants and local applications of wet cloths to the face. When apparently convalescing she was suddenly seized one morning with convulsions. These recurred frequently, her condition in the intervals being one of semi coma. She gradually sank and died. Her urine had never been albuminous. The result of the post-mortem examination was negative.

The doctor also referred to the case of the child which he had reported at a former meeting of the society. At the time of the report the child seemed to be convalescent, but soon after the disease appeared near the inner canthus of the right eye and extended over the side of the face. The constitutional symptoms were mild and the redness and swelling subsided under the application of collodion, which he supposed acted partly by protecting the surface and partly by exercising some compression. Dr. Murphy was inclined to fear that, from the number of erysipelas cases in the city, we might have an epidemic of puerperal fever.

Dr. Mackenzie stated that the first case alluded to by Dr. Murphy had been under his care upon his entering upon duty at the Hospital on the 1st. The erysipelas had entirely subsided, but the patient was quite delirious, and towards the last semi comatose. Her pulse was very weak and she was evidently sinking. She died the next day. Dr. Dandridge had carefully examined the brain and its meninges, together with the cerebral sinuses, but found nothing to account for the symptoms. He had no explanation to offer as to the cause of the delirium and convulsions in the case. He did not know anything in regard to the habits of the patient, but Dr. Murphy said he understood they were not bad.

Dr. Carson had seen the patient during life and the autopsy. He thought that there was a good deal of meningeal congestion, and that that might account for the cerebral symptoms.

Dr. Wm. Judkins referred to a case of erysipelas lately treated by him in which he had used dialyzed iron internally and carbolic acid in olive oil externally. The case seemed to do well and the patient spoke favorably of the relief given by the external application.

Dr. Davy remarked that these external applications might be of benefit by promoting the radiation of heat from the surface, and mentioned the well known case of the child which had died from the effect of an impermeable coating being applied to the whole surface; death resulting from, in all probability, the loss of heat This had also been proved experimentally in the lower animals. The application of oil to the body in cases of scarlatina might act beneficially in a similar way.

Dr. Kearney was of the opinion that external applications in erysipelas were not of much avail, except as palliatives. The disease was a self-limited one, and it is not likely that its duration would be abridged by any external application. He had not much confidence in the theory of increased radiation of heat by them, nor did he regard the tincture of the chloride of iron as at all specific, as was maintained by so many surgeons.

Dr. Murphy agreed with Dr. Kearney in believing that the beneficial effects of the tincture of chloride of iron had been greatly exaggerated. He did not think there was any specific for erysipelas; mild cases will require no treatment at all, and when the disease occurs in debilitated subjects the best remedies are tonics and stimulants.

Dr. Stanton deprecated the indiscriminate use of iron in erysipelas and also in cases of anæmia. He thought that frequently the tincture of the chloride of iron was quite injurious as producing gastric disorder, the stomach in many of the cases being rather irritable at any rate.

Dr. Walker differed from the preceding speakers in regard to iron. He relied upon it almost exclusively in erysipelas, and although he was unable to explain its mode of action, he had certainly derived benefit from it. He also used it extensively in phthisis. He supposed that erysipelas being a disease of debility, it acted as a tonic.

Dr. Murphy considered the remedy as injurious in gastric catarrh or any other disorder of the digestive functions, also when there is any tendency to hæmoptysis.

Dr. Kearney supposed that in the enormous quantities recommended by many authorities but a small quantity could be absorbed, the remainder passing out through the bowels as either inert matter or possibly after having produced by its presence irritation of the canal.

Dr. Carson thought that in many cases the excess of acid in the tincture was to be credited with the beneficial results rather than the iron.—*Cin. Lancet and Clinic.*

